# Postoperative adjuvant hepatic arterial infusion chemotherapy with gemcitabine-cisplatin sequential capecitabine combined with PDL1 inhibitors in resected high-risk intrahepatic cholangiocarcinom: study protocol for a prospective, multicenter, single-arm, phase 2 trial (HgcCP trial)

**DOI:** 10.3389/fonc.2025.1584007

**Published:** 2025-07-30

**Authors:** Ying Yang, Qing-Yun Xie, Tao Lv, Jiayin Yang, Hai-Peng Yu, Xin Zheng, Hui Zhang, Chang Liu, Hong Wu

**Affiliations:** ^1^ Department of General Surgery, West China Hospital, Sichuan University, Chengdu, China; ^2^ Liver Transplant Center, Transplant Center, West China Hospital, Sichuan University, Chengdu, China; ^3^ Department of Interventional Therapy, Tianjin Medical University Cancer Institute and Hospital, Tianjin, China; ^4^ Department of Hepatobiliary Surgery, The First Affiliated Hospital of Xi’an Jiaotong University, Xi’an, Shaanxi, China; ^5^ Department of Hepatobiliary Surgery, First Affiliated Hospital of Third Military Medical University (Army Medical University), Chongqing, China; ^6^ Division of Abdominal Tumor Multimodality Treatment, Cancer Center, West China Hospital, Sichuan University, Chengdu, Sichuan, China

**Keywords:** intrahepatic cholangiocarcinoma, adjuvant therapy, hepatic arterial infusion chemotherapy, immunotherapy, chemotherapy, phase 2 study

## Abstract

**Background:**

Although capecitabine is recommended for postoperative adjuvant treatment of biliary tract cancers (BTC), no studies have specifically focused on the postoperative adjuvant treatment of intrahepatic cholangiocarcinoma (ICC). In recent years, the combination of PD-L1 inhibitors and gemcitabine-cisplatin (GC) has demonstrated promising results in advanced BTC. The combination of GC, PD-L1 inhibitors, and capecitabine may be a potential adjuvant treatment for ICC. This phase II trial evaluates a novel regimen integrating hepatic arterial infusion chemotherapy (HAIC) with GC, sequential capecitabine, and PD-L1 inhibitors (HgcCP) for high-risk ICC after curative surgery.

**Methods:**

This multicenter, single-arm trial enrolls ICC patients underwent radical surgery. Participants receive two cycles of HAIC with GC, followed by six cycles of capecitabine and eight cycles of PD-L1 inhibitor therapy. After completion of these therapies, patients will enter a 36-month follow-up period. The primary endpoints are recurrence-free survival (RFS) and safety; secondary endpoints include overall survival (OS) and time to recurrence (TTR).

**Discussion:**

The HgcCP trial aims to establish a safe and effective adjuvant strategy for high-risk ICC after curative surgery, leveraging localized HAIC delivery and systemic immunotherapy. Results may guide future phase III trials.

**Ethics and trial registration:**

This study has been approved by the Ethics Committee of West China Hospital of Sichuan University (IRB No. 2024-1982). The trial was prospectively registered at Chinese Clinical Trial Registry (http://www.chictr.org.cn, ChiCTR2500097319) on February 17, 2025.

## 1 Introduction

Intrahepatic cholangiocarcinoma (ICC), originating from the epithelial cells of the hepatobiliary ducts, is a highly lethal malignancy with a 5-year survival rate of approximately 9% (1). It is the second most common primary liver cancer, accounting for 10%-20% cases of primary liver cancers (1, 2) and 3% of all gastrointestinal malignancies (1). Even worse, the incidence of ICC is exhibiting a significant upward trend globally (2). For instance, over the past four decades, the incidence of ICC in the United States has risen by over 140% (3). Currently, surgical resection remains the mainstay of curative treatment; however, only 20–30% of patients are eligible for resection at diagnosis (4). Even among patients who undergo radical resection, the 5-year overall survival (OS) is only 20–35% (1). The high postoperative recurrence rate, with a 3-year recurrence rate reaching up to 80% (5, 6), is the primary driver of poor long-term prognosis. Consequently, optimizing postoperative therapeutic strategies to eliminate “minimal residual disease” and mitigate recurrence risk represents a critical priority in improving long-term outcomes for high-risk ICC after curative surgery.

Several multicenter phase III trials have evaluated adjuvant therapy for biliary tract cancer (BTC). The French PRODIGE 12-ACCORD18 trial (7) randomized 196 patients with completely resected (R0/R1), non-metastatic BTC to either gemcitabine-oxaliplatin (GEMOX) or observation alone (no active adjuvant treatment). Results demonstrated no survival benefit for GEMOX versus observation. Similarly, Japan’s BCAT trial (8) compared adjuvant gemcitabine monotherapy with observation alone in resected BTC patients. This study also yielded negative outcomes: median overall survival (OS) was comparable between arms (62.3 *vs*. 63.8 months; HR 1.01, *P*= 0.964), as was recurrence-free survival (RFS) (36.0 *vs*. 39.9 months; HR 0.93, *P* = 0.693). In contrast, the BILCAP (9) and ASCOT (10) trials established positive adjuvant benefits. BILCAP showed capecitabine significantly improved both OS (51.1 *vs*. 36.4 months) and RFS (24.4 *vs*. 17.5 months) versus observation alone, with favorable tolerability. And the ASCOT (10) study also showed significant improvements in OS and RFS with adjuvant S-1 compared to observation. Consequently, NCCN guidelines (11) recommend capecitabine, while CSCO guidelines (12) endorse both capecitabine and S-1 as standard adjuvant options for BTC.

In recent years, two groundbreaking studies (13, 14) have emerged in the field of BTC immunotherapy, offering new hope for patients with advanced BTC. The TOPAZ-1 study (13) is the first phase III clinical trial to demonstrate positive results with immunotherapy combined with chemotherapy in the first-line treatment of BTC. After three years of continuous follow - up, this study confirmed that compared with the placebo + gemcitabine-cisplatin (GC) group, the median OS (mOS) in the durvalumab + GC group was significantly improved, and the patient’s overall tolerance was better. Following the TOPAZ-1 trial, the KEYNOTE - 966 study (14), with a median follow - up of 36.6 months, showed that compared with placebo + GC, pembrolizumab + GC maintained a clinically significant survival improvement in OS and the toxicity was manageable in patients with locally advanced or metastatic BTC. These two studies confirmed the clinical benefit of combining PD-1/PD-L1-targeted immunotherapy with chemotherapy for advanced BTC. Consequently, these two regimens have been recommended by the NCCN (11) and CSCO (12) guidelines for the treatment of advanced BTC. Additionally, the subgroup analysis of both studies showed that the ICC patients benefited more significantly from immunotherapy combined with chemotherapy.

Compared with systemic therapy (SYS), HAIC can significantly enhance antitumor activity while reducing damage to normal liver tissue. In recent years, the combination of HAIC and SYS has shown promising results in the treatment of BTC. For instance, a propensity score-matching analysis evaluated the efficacy of SYS with or without HAIC in ICC patients with extrahepatic oligometastasis. Results indicated that HAIC plus SYS could significantly improve OS (15.8 months *vs*. 12.7 months, *P* = 0.023) and intrahepatic PFS (9.7 months *vs*. 6.1 months, *P* < 0.001) compared with SYS alone, and the combination therapy had a significantly lower mortality rate due to liver failure (15). In another single-center retrospective study (16) involving 41 patients with unresectable BTC, the results showed that combination therapy with HAIC-FOLFOX (oxaliplatin, fluorouracil and leucovorin) and lenvatinib significantly improved both OS and PFS compared with HAIC-FOLFOX alone. In addition, HAIC combined with immunotherapy for BTC also demonstrated a favorable therapeutic effect. For example, a study aimed to compare the efficacy and safety of HAIC plus Lenvatinib with or without Programmed Death - 1 Inhibitor (PD - 1i) in patients with advanced Cholangiocarcinoma (CCA). Fifty-five patients with advanced CCA were included and divided into two groups, with the HAIC + Lenvatinib + PD - 1i group (n = 35) and the HAIC + Lenvatinib group (n = 20). After a median follow-up time of 14.0, the PFS and OS of patients in the HAIC + lenvatinib + PD-1i group were significantly better than those in the HAIC + lenvatinib group (17). Similarly, a retrospective study (18) included 46 patients with locally advanced or metastatic ICC who received triple therapy with Lenvatinib, PD - 1 inhibitor and HAIC_FOLFOX. The primary endpoints showed that the median PFS was 9.40 months, the 6 - month PFS rate was 76.1%. Another study (17) evaluated the efficacy and safety of Lenvatinib plus Durvalumab combined with HAIC_FOLFOX for unresectable ICC. The results showed an objective response rate (ORR) based on mRECIST and RECIST 1.1 criteria were 65.2% and 39.1% respectively. The median OS was 17.9 months, and the median PFS was 11.9 months. Among the 28 enrolled patients, 23 patients could undergo tumor remission evaluation. The incidence of grade 3–4 adverse events (AEs) was 46.5% (13/28). Additionally, a retrospective study (19) evaluated another combination therapy (HAIC_GEMOX) plus systemic Gemcitabine chemotherapy (GEM - SYS) combined with Lenvatinib and PD - 1i for patients with large unresectable ICC (uICC). After 16 months of follow - up, the results showed that this combination therapy was effective for these patients, with a median OS of 19.5 months and a median PFS of 6.0 months, and was well tolerated with no grade 5 AEs reported. Overall, these studies suggest that the combination of HAIC and SYS can improve the outcomes of cholangiocarcinoma patients compared with either SYS alone or HAIC alone, representing a more promising treatment approach.

However, most studies do not specifically address postoperative adjuvant treatment for ICC. Many of these studies enroll patients with other types of BTC; whether the results are applicable to ICC patients remains to be further discussed. Moreover, most studies on ICC are based on small-sample retrospective studies and meta-analyses of SEER data, lacking the robust evidence provided by large-scale prospective randomized controlled trials (RCTs) (2). Therefore, we designed a Phase II study to evaluate the efficacy and safety of HAIC with GC followed by capecitabine combined with PDL1 inhibitors (HgcCP) as the postoperative adjuvant treatment for high-risk ICC after curative surgery.

## 2 Methods and design

**Figure 1:**
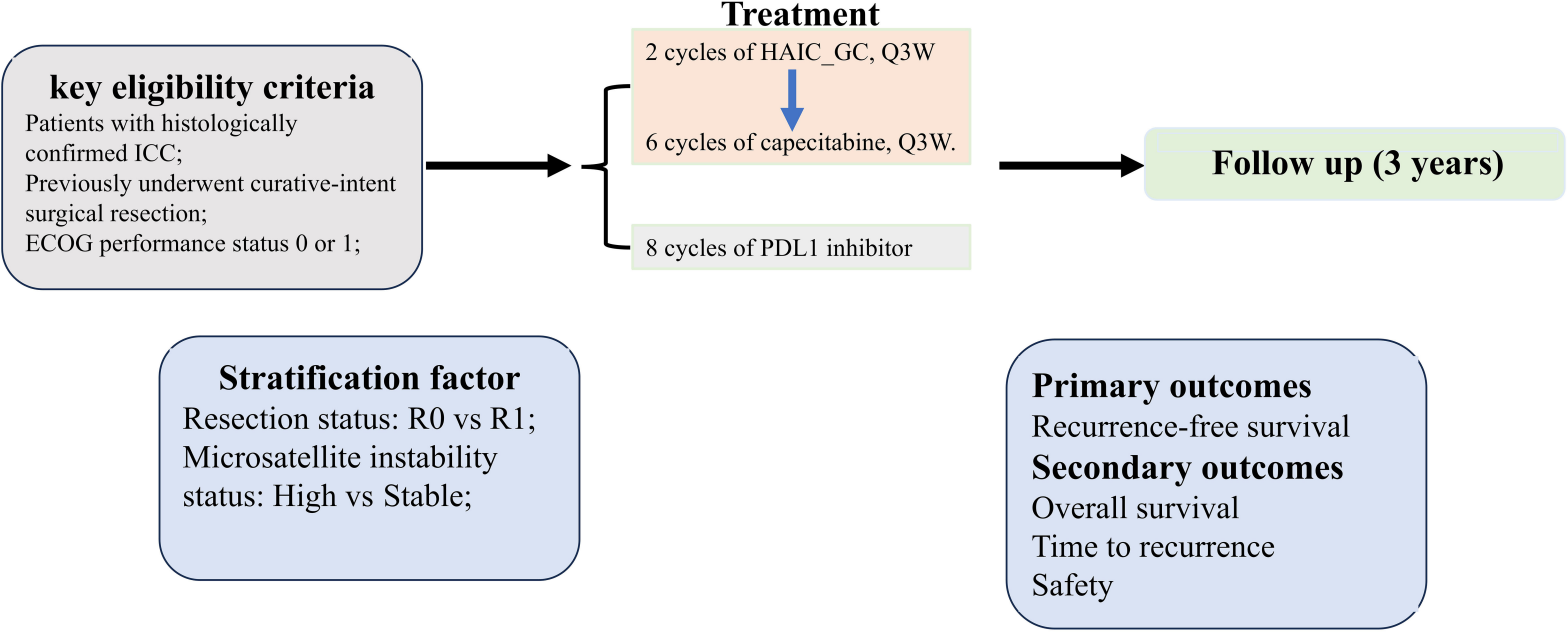
Flowchart of the study protocol.

This investigator-initiated phase II trial employs a multicenter, single-arm design to assess the safety and efficacy of the HgcCP regimen as an adjuvant therapy for high-risk ICC after curative surgery. Enrollment is scheduled from March 2025 to March 2027, with a 36-month follow-up period initiated after the last participant’s inclusion. While acknowledging potential biases in single-arm trials, we implemented: (1) blinded Independent Review Facility (IRF) adjudication of RFS; (2) propensity score-adjusted sensitivity analysis against historical ICC controls; (3) transparent screening logs documenting exclusion reasons. The protocol structure and assessment timelines are detailed in **Figure 1** and **Table 1**, respectively.

**Table 1:** Schedule of clinical assessments for enrolled patients.

Assessments	Pre-enrollment baseline (≤7 days)	Cycle initiation ^c^	Post-Treatment Evaluation (≤4 weeks after final PD-L1 inhibitor dose)	Follow-Up Schedule
Enrollment: Eligibility screen ^a^ and Informed consent ^b^	✓			
Baseline characteristics^#^	✓			
Pathological assessment^#^	✓			
Laboratory Tests^#^	✓	✓	✓	✓ (Year 1: Quarterly; Years 2–3: Biannual)
Imaging examination^#^	✓	✓	✓	✓ (Year 1: Quarterly; Years 2–3: Biannual)
Toxicity/Adverse Event monitoring		✓	✓	✓ (Year 1: Quarterly; Years 2–3: Biannual)
Quality of Life	✓	✓	✓	✓ (Year 1: Quarterly; Years 2–3: Biannual)
Relapse		✓	✓	✓ (Year 1: Quarterly; Years 2–3: Biannual)
Survival		✓	✓	✓ (Year 1: Quarterly; Years 2–3: Biannual)

a The criteria for screening eligibility can be found in the subsequent section on patient selection.

b The informed consent form is provided in both the native language (Chinese) and the corresponding English version, which can be found in **Supplementary Material 1**.

c The time arrangement of the first two cycles will be implemented in accordance with the cycle of HAIC. After the completion of HAIC treatment, the subsequent treatment cycles will be based on the treatment cycle of the PDL1 inhibitor.

^#^Specific items should refer to the subsequent data collection.

### 2.1 Participant eligibility

Eligible participants must meet the following key criteria: pathologically confirmed ICC; have had radical surgical treatment; age 18–75 years; life expectancy ≥3 months; Eastern Cooperative Oncology Group (ECOG) score ≤1; and adequate hematologic, hepatic, and renal function. Comprehensive inclusion/exclusion criteria are summarized in **Table 2**.

**Table 2:** Study eligibility criteria.

Inclusion criteria	Exclusion criteria
1. Patients with histologically confirmed ICC	1. Active malignancy within 5 years (excluding non-melanoma skin/cervical carcinoma *in situ*)
2. Previously underwent curative-intent surgical resection	2. Use of iodine and gadolinium contraindicated due to allergic to contrast agents.
3. Aged 18 to 75 years, regardless of gender	3. Abnormalities of the hepatic artery prevent the implementation of HAIC.
4. ECOG performance status 0 or 1	4. Pregnancy, lactation, or refusal of contraception
5. Expected life expectancy of ≥ 3 months	5. Previous treatment with immunotherapy, for instance anti-PD-1, anti-PD-L1, anti-PD-L2, or any other T-cell co-stimulation or checkpoint inhibitor therapy
6. Confirm the absence of extrahepatic spread and tumor recurrence through CT or MRI scans of the chest, abdomen, pelvis, and head.	6. A history of gastrointestinal bleeding within 3 months prior to enrollment;
7. Between 4 and 12 weeks after resection. Have recovered to a fully rehabilitated state from surgical resection: adequate wound healing after the operation, with the surgical drain removed.	7. A history of arterial and venous thrombotic events within 6 months prior to enrollment, including myocardial infarction, cerebrovascular accidents vein thrombosis and pulmonary embolism;
8. Adequate oral intake.	8. A history of substance or psychotropic substance abuse;
9. Adequate renal functions: as follows: serum creatine<1.5-times upper limit of normal (ULN); Calculated glomerular filtration rate (GFR) ≥60 ml/min.	9. Underwent organ transplantation before
10. Adequate hematological function: neutrophil count≥1.5×10^9^/L; hemoglobin≥80g/L; platelet count≥60×10^9^/L; WBC≥3.0 x 109/L	10. Hereditary or acquired bleeding and thrombotic tendencies, such as hemophilia, coagulation disorders, thrombocytopenia, hypersplenism, etc.;
11. Adequate liver function: serum albumin≥28g/L; total bilirubin<3-times ULN; ALT<5-times ULN; AST<5-times ULN;	11. be allergic to related drugs
12. The Child-Pugh score is between 5 and 7.	12. Any previous chemotherapy or immunotherapy, given for ICC.
13. Voluntarily participate and sign an informed consent.	13. Any serious uncontrolled medical conditions likely to interfere with protocol treatment.
14. High risk for ICC recurrence after resection ^a^	

a High-risk features for resected patients include patients with greater than or equal to stage T2 (20, 21), low tumor differentiation (5, 22), presence of multiple tumors (at least 3 lesions) or satellite nodules (5), tumor size exceeding 5 cm (22), (microvascular) vascular and neural invasion (5, 23).

### 2.2 Treatment

Two cycles of HAIC_GC and then six cycles of capecitabine will be administered. Meanwhile, the PDL1 inhibitor will be given on a 21-day cycle for up to eight cycles.

1. Two cycles of HAIC_GC: cisplatin 25mg/m² on the first day, with arterial infusion for half an hour; gemcitabine 1000mg/m² on the first day, with arterial infusion for half an hour. Repeat every 3 weeks for 2 cycles of treatment.
2. Six cycles of capecitabine: 1250 mg/m^2^, taken orally, twice a day, continuously for 14 days. One cycle lasts for 21 days, with a total of six cycles.
3. Eight cycles of PDL1 inhibitor (Adebrelimab): 20 mg/kg, administered intravenously on day one of each cycle. Repeat every 3 weeks for 8 cycles of treatment.

Throughout the entire trial period, any potential AEs will undergo rigorous surveillance by the Ethics Review Committee of West China Hospital, Sichuan University. All participants are explicitly informed of their unrestricted right to withdraw from the study at any point.

### 2.3 Criteria for study discontinuation

The trial should be stopped immediately in the following cases:

1. The patient requests to terminate the trial, the Ethics Committee demands to terminate the trial, or the drug regulatory department requires to terminate the trial.
2. The investigators confirm that the disease has progressed or that continuing treatment would lack therapeutic benefit.
3. Participant- or clinician-reported unacceptable AEs.
4. The patient fails to strictly adhere to the treatment protocol.

All withdrawal rationales will be systematically documented in the trial’s clinical records.

### 2.4 Data collection

1. Baseline patient characteristics:

- Age, year
- Gender, male/female
- Body mass index, kg/m^2^
- Personal history of smoking, alcohol intake, and medication usage
- Comorbidities
- ECOG performance status
- Child-Pugh class
- Hepatitis B status
- Hepatitis C status

2. Laboratory examination

- CA19-9, UI/mL
- CEA, ng/mL
- Hemoglobin (g/dL)
- White blood cell count, × 10^9^ cells per L
- Absolute neutrophil count, × 10^9^ cells per L
- Platelet count, × 10^9^ per L
- Glomerular filtration rate, mL/min
- Aspartate aminotransferase, U/L
- Alanine aminotransferase, U/L
- Bilirubin, μmol/L
- Creatinine, μmol/L
- thyroid function indices

3. Imaging examination

- liver MRI/CT scans,
- chest CT scans
- isotope bone scans
- liver ultrasound
- electrocardiogram results

4. Pathological assessment

- Largest tumor diameter, cm
- Grade of differentiation, including Well differentiated, Moderately differentiated, Poorly differentiated, Not specified.
- AJCC 8th edition T stage (*AJCC*, American Joint Committee on Cancer)
- AJCC 8th edition N stage
- Resection status, including R0 and R1
- Microsatellite instability (MSI) status, including High, Stable and Missing (Missing MSI status encompasses cases with undetermined results or absence of testing.)

### 2.5 Outcome measures

1. Primary outcomes:

- RFS, RFS is calculated as the interval between trial enrollment and the earliest recorded event, including locoregional recurrence, metastatic progression of ICC, or all-cause mortality and determined by an IRF
- Safety:

- Incidence of AEs: AEs will be systematically classified and documented in compliance with National Cancer Institute’s Common Terminology Criteria for Adverse Events Version 5.0 (CTCAE 5.0) (24)(**Supplementary Material 2**).
- Incidence of HAIC complications: The time frame is within three months after HAIC;
- Functional assessment of cancer therapy-general (FACT-G) (25)

2. Secondary outcomes:

- OS, the interval from study enrollment to mortality from any cause.
- Time to recurrence (TTR), TTR is calculated as the interval between trial enrollment and the first recurrence, evaluated by both the investigator and by an IRF.

### 2.6 Sample size

**Figure 2:**
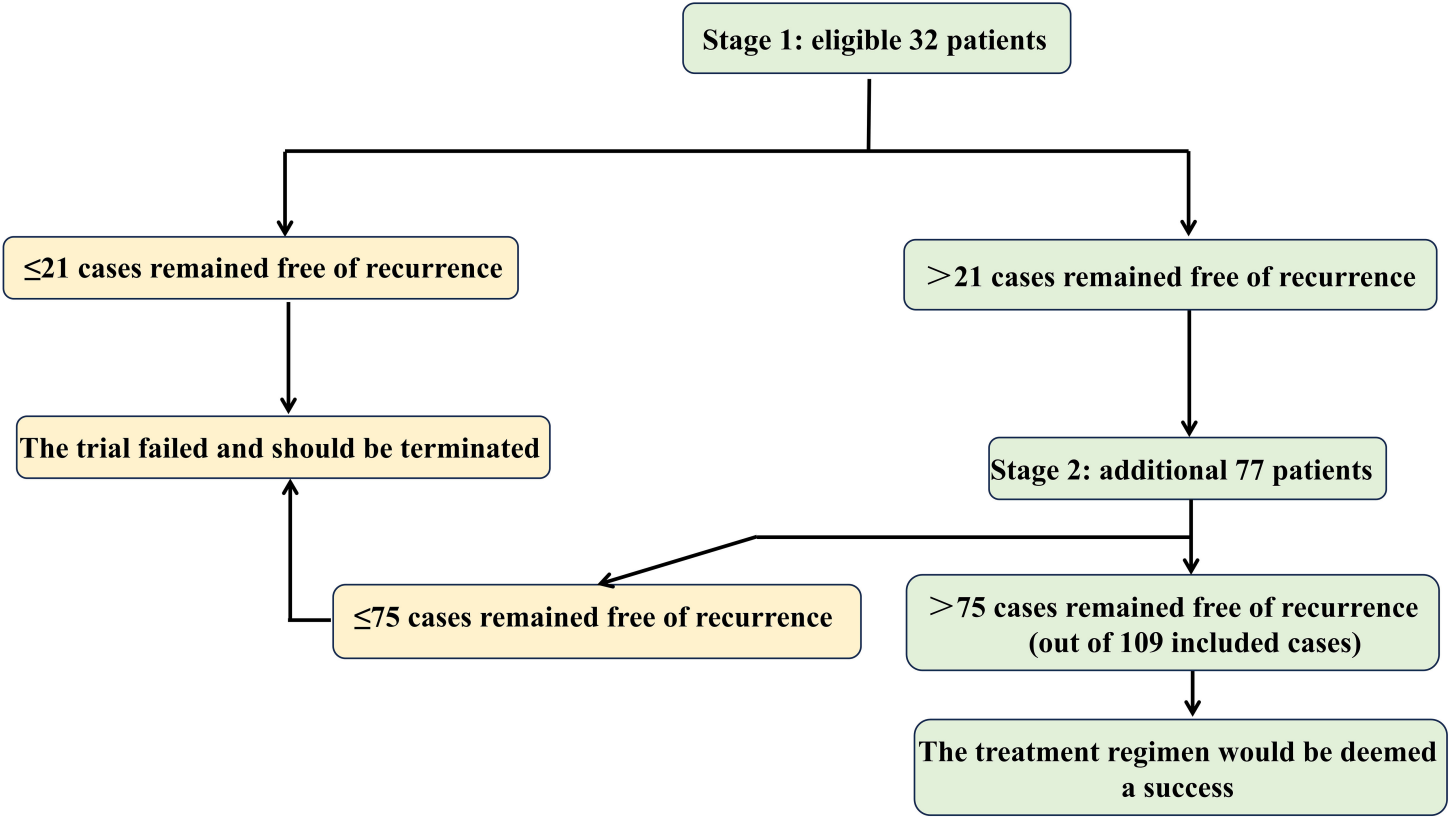
Schematic of Simon’s two-stage design for sample size calculation in the HgcCP trial.

Sample size was determined using Simon’s optimal two-stage design (26) (**Figure 2**), implemented in PASS software with the optimal criterion to minimize expected sample size under H_0_. Key parameters were: (1) Type I error (α): 0.05 (one-sided), standard for phase II efficacy trials; (2) Type II error (β): 0.20 (power = 80%); (3) Reference values: 1) Null hypothesis (H_0_): 3-year RFS = 62.4% (derived from ASCOT’s BTC cohort (10))); 2) Alternative hypothesis (H_1_): 3-year RFS = 75.0% (targeting clinically meaningful improvement in high-risk ICC). The first stage required 32 patients and if more than 21 cases remained free of local, regional or metastatic ICC or death during the 3-year follow-up, the second stage would recruit 77 additional participants, yielding a total cohort of 109. To address a projected 10% attrition rate, the sample size should be increased accordingly. Therefore, a total of 121 subjects will be recruited in this study.

### 2.7 Data management and monitoring plan

Data collection will be conducted using a validated case report form (CRF) by trained evaluators, ensuring timeliness, completeness, and accuracy. Two independent data-entry staffs will independently receive the completed CRFs and input the raw data into Epidata (V3.1). An independent monitoring Committee—comprising multidisciplinary experts in oncology, interventional radiology, pharmacology, and biostatistics—will oversee trial data integrity and protocol adherence. They possess the prerogative to halt the trial prematurely if necessary. Following study closure, all CRFs will undergo archival storage for a minimum of five years, with read-only access implemented to prevent *post-hoc* alterations.

### 2.8 Statistical analysis

The primary endpoint is the RFS of patients, which will be analyzed based on the intention-to-treat (ITT) population. All variables will be described using applicable descriptive statistics. Measurement data will be expressed as mean values and standard deviation or median and interquartile range (for skewed distribution). And discrete data will be presented as frequency and percentage distributions. The RFS and OS were calculated using Kaplan-Meier method. Univariate and multivariate survival analyses will be performed using Cox regression analysis to calculate hazard ratios (HRs) and 95% confidence intervals (CIs). All statistical analyses will be performed using SPSS V.22 software or R software V.4.3.1 (http://www.R-project.org), with statistical significance defined as a two-tailed *p* < 0.05.

### 2.9 Confidentiality

All research - related data will be stored in a highly secure environment at the study site. Participants’ personal information will be safeguarded in encrypted digital databases with restricted access. To maintain participant confidentiality, any identifiable data such as laboratory results, questionnaires, data analysis outcomes, and administrative records will be labeled solely with anonymous identification codes.

### 2.10 Patient and public involvement

No participants or the public will be involved in the protocol development and study design. Results of this study will be presented at peer-reviewed journals or international meetings.

## 3 Discussion

ICC ranks among the most prevalent malignancies globally. While radical resection offers the primary curative intent, over 80% of patients experience tumor relapse within 3 years after surgery (5, 6), underscoring the critical need for effective adjuvant strategies. Two clinical trials (9, 10) have confirmed that postoperative capecitabine or S-1(tegafur/gimeracil/oteracil) treatment could improve the OS and RFS of patients with BTC. Emerging evidence from these two studies supports the adoption of multimodal therapy—integrating radical resection and systemic chemotherapy—as the first-line regimen for BTC management. In fact, the subjects included in these two studies were not only ICC patients. And a study (27) analyzing the molecular characteristics of BTC showed that there was great heterogeneity in the pathogenesis between ICC and extrahepatic cholangiocarcinoma. However, there are hardly any studies on postoperative adjuvant treatment that only include ICC patients as the research subjects. Therefore, this single-arm, multicenter prospective phase II study was designed to assess the safety and efficacy of HgcCP as adjuvant therapy for high-risk ICC following curative resection, aiming to address the unmet need for optimized postoperative strategies in these patients.

The HgcCP adjuvant strategy integrates three evidence-based therapeutic approaches: (1) Immunochemotherapy synergy: TOPAZ-1 (13) and KEYNOTE-966 (14) established survival benefits with PD-L1 inhibitors combined with GC in advanced BTC, with ICC subgroups demonstrating superior outcomes (TOPAZ-1: OS HR=0.79, 95%CI:0.64∼0.99; KEYNOTE-966:OS HR=0.76, 95%CI:0.64∼0.91); (2) Guideline-directed adjuvant therapy: capecitabine remains the standard postoperative treatment for resected BTC based on the BILCAP (9) and ASCOT study (10); (3) Locoregional optimization: HAIC enhances intrahepatic drug delivery while minimizing systemic toxicity (15–18), particularly relevant given ICC’s liver-dominant recurrence pattern. Our sequential design—initial HAIC-delivered GC followed by capecitabine plus PD-L1 inhibition—simultaneously leverages HAIC’s cytoreductive effect on micrometastases while mitigating synergistic toxicities. To our knowledge, this represents the first phase II trial investigating this triple-modality approach (HAIC-GC and sequential capecitabine/PD-L1 inhibition) as adjuvant therapy for resected high-risk ICC.

One limitation of this study is that, due to the single-arm design, biases may exist in patient selection and outcome assessment. However, this approach was necessitated by the rarity of high-risk ICC after curative surgery and ethical constraints against inactive controls. Our pre-planned comparative analyses with propensity score adjustment partially mitigate this limitation. And this study may yield exciting results and lay the foundation for the subsequent Phase III clinical trials. In addition, this clinical trial will be carried out in multiple clinical centers. Therefore, the predefined sample size is relatively easy to obtain, thereby enhancing the statistical reliability of the study outcomes.

## Data Availability

The original contributions presented in the study are included in the article/**Supplementary Material**. Further inquiries can be directed to the corresponding author.

## References

[1] Moris D Palta M Kim C Allen PJ Morse MA Lidsky ME . Advances in the treatment of intrahepatic cholangiocarcinoma: An overview of the current and future therapeutic landscape for clinicians. CA: Cancer J Clin. (2023) 73:198–222. doi: 10.3322/caac.21759, PMID: 36260350

[2] Vogel A Bridgewater J Edeline J Kelley RK Klümpen HJ Malka D . Biliary tract cancer: ESMO Clinical Practice Guideline for diagnosis, treatment and follow-up. Ann Oncol. (2023) 34:127–40. doi: 10.1016/j.annonc.2022.10.506, PMID: 36372281

[3] Saha SK Zhu AX Fuchs CS Brooks GA . Forty-year trends in cholangiocarcinoma incidence in the U.S.: intrahepatic disease on the rise. oncologist. (2016) 21:594–9. doi: 10.1634/theoncologist.2015-0446, PMID: 27000463 PMC4861366

[4] Endo I Gonen M Yopp AC Dalal KM Zhou Q Klimstra D . Intrahepatic cholangiocarcinoma: rising frequency, improved survival, and determinants of outcome after resection. Ann surg. (2008) 248:84–96. doi: 10.1097/SLA.0b013e318176c4d3, PMID: 18580211

[5] Mavros MN Economopoulos KP Alexiou VG Pawlik TM . Treatment and prognosis for patients with intrahepatic cholangiocarcinoma: systematic review and meta-analysis. JAMA surg. (2014) 149:565–74. doi: 10.1001/jamasurg.2013.5137, PMID: 24718873

[6] Tsilimigras DI Sahara K Wu L Moris D Bagante F Guglielmi A . Very early recurrence after liver resection for intrahepatic cholangiocarcinoma: considering alternative treatment approaches. JAMA surg. (2020) 155:823–31. doi: 10.1001/jamasurg.2020.1973, PMID: 32639548 PMC7344787

[7] Edeline J Benabdelghani M Bertaut A Watelet J Hammel P Joly JP . Gemcitabine and oxaliplatin chemotherapy or surveillance in resected biliary tract cancer (PRODIGE 12-ACCORD 18-UNICANCER GI): A randomized phase III study. J Clin Oncol. (2019) 37:658–67. doi: 10.1200/jco.18.00050, PMID: 30707660

[8] Ebata T Hirano S Konishi M Uesaka K Tsuchiya Y Ohtsuka M . Randomized clinical trial of adjuvant gemcitabine chemotherapy versus observation in resected bile duct cancer. Br J surg. (2018) 105:192–202. doi: 10.1002/bjs.10776, PMID: 29405274

[9] Primrose JN Fox RP Palmer DH Malik HZ Prasad R Mirza D . Capecitabine compared with observation in resected biliary tract cancer (BILCAP): a randomised, controlled, multicentre, phase 3 study. Lancet Oncol. (2019) 20:663–73. doi: 10.1016/s1470-2045(18)30915-x, PMID: 30922733

[10] Ikeda M Nakachi K Konishi M Nomura S Katayama H Kataoka T . Adjuvant S-1 versus observation in curatively resected biliary tract cancer: A phase III trial (JCOG1202: ASCOT). J Clin Oncol. (2022) 40:382–2. doi: 10.1200/JCO.2022.40.4_suppl.382

[11] Benson AB D’Angelica MI Abrams T Abbott DE Ahmed A Anaya DA . NCCN guidelines^®^ Insights: biliary tract cancers, version 2.2023. J Natl Compr Cancer Network: JNCCN. (2023) 21:694–704. doi: 10.6004/jnccn.2023.0035, PMID: 37433432

[12] Wang X Bai Y Chai N Li Y Linghu E Wang L . Chinese national clinical practice guideline on diagnosis and treatment of biliary tract cancers. Chin Med J. (2024) 137:2272–93. doi: 10.1097/cm9.0000000000003258, PMID: 39238075 PMC11441919

[13] Burris HA 3rd Okusaka T Vogel A Lee MA Takahashi H Breder V . Durvalumab plus gemcitabine and cisplatin in advanced biliary tract cancer (TOPAZ-1): patient-reported outcomes from a randomised, double-blind, placebo-controlled, phase 3 trial. Lancet Oncol. (2024) 25:626–35. doi: 10.1016/s1470-2045(24)00082-2, PMID: 38697156

[14] Kelley RK Ueno M Yoo C Finn RS Furuse J Ren Z . Pembrolizumab in combination with gemcitabine and cisplatin compared with gemcitabine and cisplatin alone for patients with advanced biliary tract cancer (KEYNOTE-966): a randomised, double-blind, placebo-controlled, phase 3 trial. Lancet (London England). (2023) 401:1853–65. doi: 10.1016/s0140-6736(23)00727-4, PMID: 37075781

[15] Li Z Xu R Chang X Sun P . Systemic chemotherapy with or without hepatic arterial infusion chemotherapy for intrahepatic cholangiocarcinoma with extrahepatic oligometastasis: A propensity score-matched analysis. J Vasc interventional radiol: JVIR. (2024) 35:416–27.e417. doi: 10.1016/j.jvir.2023.11.015, PMID: 38008375

[16] Wang Y Wei Z Zhang Z Xu J Wang Y Chen Q . Hepatic arterial infusion chemotherapy with or without lenvatinib for unresectable cholangiocarcinoma: a single-center retrospective study. Hepatic Oncol. (2023) 10:Hep49. doi: 10.2217/hep-2023-0006, PMID: 37850031 PMC10577516

[17] Zhao R Zhou J Miao Z Xiong X Wei W Li S . Efficacy and safety of lenvatinib plus durvalumab combined with hepatic arterial infusion chemotherapy for unresectable intrahepatic cholangiocarcinoma. Front Immunol. (2024) 15:1397827. doi: 10.3389/fimmu.2024.1397827, PMID: 38799453 PMC11116590

[18] Huang Y Du Z Kan A He M Li H Lai Z . Clinical and biomarker analyses of hepatic arterial infusion chemotherapy plus lenvatinib and PD-1 inhibitor for patients with advanced intrahepatic cholangiocarcinoma. Front Immunol. (2024) 15:1260191. doi: 10.3389/fimmu.2024.1260191, PMID: 38384459 PMC10880187

[19] Ni JY Sun HL Guo GF Zhou X Wei JX Xu LF . Hepatic arterial infusion of GEMOX plus systemic gemcitabine chemotherapy combined with lenvatinib and PD-1 inhibitor in large unresectable intrahepatic cholangiocarcinoma. Int immunopharmacol. (2024) 140:112872. doi: 10.1016/j.intimp.2024.112872, PMID: 39121605

[20] Reames BN Bagante F Ejaz A Spolverato G Ruzzenente A Weiss M . Impact of adjuvant chemotherapy on survival in patients with intrahepatic cholangiocarcinoma: a multi-institutional analysis. HPB. (2017) 19:901–9. doi: 10.1016/j.hpb.2017.06.008, PMID: 28728891

[21] Li XH Zhao CY Zhou EL Lin XJ . Efficacy and safety of adjuvant chemotherapy in T1N0M0 intrahepatic cholangiocarcinoma after radical resection. BMC cancer. (2022) 22:1159. doi: 10.1186/s12885-022-10269-0, PMID: 36357848 PMC9650851

[22] Sun Y Jiang W Duan R Guan L . Research progress and prospect of postoperative adjuvant therapy for resectable intrahepatic cholangiocarcinoma. Front Pharmacol. (2024) 15:1432603. doi: 10.3389/fphar.2024.1432603, PMID: 39170710 PMC11335543

[23] Wei T Zhang XF He J Popescu I Marques HP Aldrighetti L . Prognostic impact of perineural invasion in intrahepatic cholangiocarcinoma: multicentre study. Br J surg. (2022) 109:610–6. doi: 10.1093/bjs/znac098, PMID: 35511599

[24] Dueck AC Mendoza TR Mitchell SA Reeve BB Castro KM Rogak LJ . Validity and reliability of the US national cancer institute’s patient-reported outcomes version of the common terminology criteria for adverse events (PRO-CTCAE). JAMA Oncol. (2015) 1:1051–9. doi: 10.1001/jamaoncol.2015.2639, PMID: 26270597 PMC4857599

[25] Cella DF Tulsky DS Gray G Sarafian B Linn E Bonomi A . The Functional Assessment of Cancer Therapy scale: development and validation of the general measure. J Clin Oncol. (1993) 11:570–9. doi: 10.1200/jco.1993.11.3.570, PMID: 8445433

[26] Simon R . Optimal two-stage designs for phase II clinical trials. Controlled Clin trials. (1989) 10:1–10. doi: 10.1016/0197-2456(89)90015-9, PMID: 2702835

[27] Spencer K Pappas L Baiev I Maurer J Bocobo AG Zhang K . Molecular profiling and treatment pattern differences between intrahepatic and extrahepatic cholangiocarcinoma. J Natl Cancer Institute. (2023) 115:870–80. doi: 10.1093/jnci/djad046, PMID: 37040087 PMC10323903

