# Supplementary material for: Postoperative adjuvant hepatic arterial infusion chemotherapy with gemcitabine-cisplatin sequential capecitabine combined with PDL1 inhibitors in resected high-risk intrahepatic cholangiocarcinom: study protocol for a prospective, multicenter, single-arm, phase 2 trial (HgcCP trial)
Source: Front Oncol. 2025 Jul 30;15:1584007. doi: 10.3389/fonc.2025.1584007 (PMC12343247; doi:10.3389/fonc.2025.1584007)
Supplement: Supplementary file 1 [file DataSheet1.docx]

**West China Hospital, Sichuan University**

**Participant Informed Consent**

We invite you to participate in the research of "Postoperative Adjuvant Hepatic Arterial Infusion Chemotherapy With Gemcitabine-Cisplatin Sequential Capecitabine Combined With PDL1 inhibitors in Intrahepatic Cholangiocarcinom: Study Protocol for An Investigator-initiated Phase 2 Trial (HgcCP trial)", approved by West China Hospital of Sichuan University. It is estimated that 121 subjects will participate voluntarily. This study has been reviewed and approved by the Biomedical Ethics Committee of West China Hospital of Sichuan University.

1. **Why should we carry out this study?**

Intrahepatic cholangiocarcinoma (ICC), originating from the epithelial cells within the hepatobiliary ducts, is an almost universally lethal malignancy which 5-year survival rates are only about 9%. It is the second most common primary liver cancer, accounting for 10%-20% cases of primary liver cancers and 3% of all gastrointestinal malignancies. Even worse, the incidence of ICC is still showing a significant upward trend worldwide. For instance, over the past four decades, the incidence of ICC has increased by more than 140% in the USA. At present, surgical resection remains as the mainstay of curative treatment option, however, only 20 - 30% of patients are considered suitable for resection at the time of diagnosis. In fact, even for ICC patients who have undergone radical resection, the 5-year overall survival (OS) is only 20 - 35%. The persistently high postoperative recurrence rate, with a 3-year recurrence rate as high as 80%, is the main reason for the poor long-term prognosis. Therefore, there is an urgent need for postoperative adjuvant therapies to eradicate “minimal residual disease” and to prevent postsurgical relapse.

Currently, the guidelines recommend using capecitabine or tegafur/gimeracil/oteracil potassium as postoperative adjuvant therapy for patients with Biliary Tract Cancer (BTC). In recent years, immune checkpoint inhibitors targeting PD-1 and PDL1 combined with chemotherapy have been proven to be effective and safe in the treatment of advanced BTC. In addition, hepatic artery infusion chemotherapy (HAIC), a drug delivery method that can enhance antitumor activity while reducing systemic side effects, has been confirmed in many studies to improve the prognosis of patients with advanced BTC when combined with systemic treatment. Although ICC patients were included in the above studies, in fact, no studies focus on postoperative adjuvant treatment of patients with ICC have been reported. Whether these research conclusions are also applicable to patients with ICC still needs to be confirmed by high-quality studies. Therefore, this study is designed as a prospective single-arm phase II study to fill the research gap in the field of postoperative adjuvant treatment for ICC. It aims to explore the efficacy and safety of hepatic arterial infusion chemotherapy (HAIC) with GC plus PDL1 inhibitors and capecitabine (HAIC_GC+PDL1+ capecitabine, HgcPC) as postoperative adjuvant treatment for patients with ICC.

**2. What do you need to do if you take part in the study?**

Two cycles of HAIC_GC and then six cycles of capecitabine will be administered. Meanwhile, the PDL1 inhibitor will be given on a 21-day cycle for up to eight cycles.

1. Two cycles of HAIC_GC: cisplatin 25mg/m² on the first day, with arterial infusion for half an hour; gemcitabine 1000mg/m² on the first day, with arterial infusion for half an hour. Repeat every 3 weeks for 2 cycles of treatment.
2. Six cycles of capecitabine: 1250 mg/m^2^, taken orally, twice a day, continuously for 14 days. One cycle lasts for 21 days, with a total of six cycles.
3. Eight cycles of PDL1 inhibitor: Conduct treatment according to the corresponding instructions.

In addition, you will be enrolled, treated and followed up according to the following table1.

Table 1. Schedule of clinical assessments for enrolled patients

| Assessments | Baseline  within 1 week  prior to  enrollment | Day 1  of every  cycle ^c^ | End of eight -cycle  treatment  (within 4  weeks of last  administered  PDL1 inhibitor) | Follow-up  post-  enrollment  3 monthly  (1st year) | Follow-up  post-  enrollment  6 monthly  (2-3 year) |
| --- | --- | --- | --- | --- | --- |
| Enrollment: Eligibility screen ^a^ and Informed consent ^b^ | x |  |  |  |  |
| Baseline patient characteristics^#^ | x |  |  |  |  |
| Pathological assessment^#^ | x |  |  |  |  |
| Laboratory examination^#^ | x | x | x | x | x |
| Imaging examination^#^ | x | x | x | x | x |
| Toxicity / Adverse  Event monitoring |  | x | x | x | x |
| Quality of Life | x | x | x | x | x |
| Relapse |  | x | x | x | x |
| Survival |  | x | x | x | x |

**3. What are the treatment options available?**

Current guidelines recommend capecitabine or tegafur/gimeracil/oteracil potassium for postoperative adjuvant therapy in patients with BTC. Therefore, you can choose either capecitabine or tegafur/gimeracil/oteracil potassium for treatment.

**4. Who should not be included in the study?**

(1) Known history of other malignancy within 5 years of trial entry, except adequately treated cervical carcinoma - in - situ or non - melanotic skin cancer.

(2) Use of iodine and gadolinium contraindicated due to allergic to contrast agents.

(3) Abnormalities of the hepatic artery prevent the implementation of HAIC.

(4) Pregnant or lactating women, unwillingness to use contraceptive measures in males and females.

(5) Previous treatment with immunotherapy, for instance anti - PD - 1, anti - PD - L1, anti - PD - L2, or any other T - cell co - stimulation or checkpoint inhibitor therapy.

(6) A history of gastrointestinal bleeding within 3 months prior to enrollment.

(7) A history of arterial and venous thrombotic events within 6 months prior to enrollment, such as myocardial infarction, cerebrovascular accidents (including transient ischemic attacks, cerebral hemorrhage, cerebral infarction), deep vein thrombosis and pulmonary embolism.

(8) A history of psychotropic drug abuse or drug abuse.

(9) Underwent organ transplantation before.

(10) Hereditary or acquired bleeding and thrombotic tendencies, such as hemophilia, coagulation disorders, thrombocytopenia, hypersplenism, etc.

(11) Be allergic to related drugs.

(12) Any previous chemotherapy or immunotherapy, given for ICC.

(13) Any serious uncontrolled medical conditions likely to interfere with protocol treatment.

**5. What are the risks of participating in the study?**

1. **Adverse Drug Reactions**
2. The chemotherapy drugs (such as gemcitabine - cisplatin, capecitabine) used in the study may cause a variety of adverse reactions. For example, gemcitabine and cisplatin may cause nausea, vomiting, hair loss, myelosuppression (including leukopenia, thrombocytopenia, anemia, etc.), which can affect the patient's quality of life. In severe cases, it may lead to complications such as infection and bleeding, and even endanger life. Capecitabine may cause hand - foot syndrome, gastrointestinal reactions, and hematological toxicity.
3. PDL1 inhibitors may trigger immune - related adverse events, such as immune - related pneumonia, immune - related hepatitis, thyroid dysfunction, rash, diarrhea, etc. These adverse reactions may require close monitoring and timely treatment, and severe immune - related adverse events may pose a serious threat to the patient's health.
4. **Risks Related to HAIC**

The HAIC operation itself has certain risks, such as possible hepatic artery injury, bleeding, infection and other complications. In addition, HAIC may affect the normal blood supply of the liver, leading to liver function impairment and even liver failure.

1. **Potential Risks of Combined Treatment**

Due to the combination of multiple drugs and treatment methods in this study, the incidence and severity of adverse reactions may increase. For example, the combination of chemotherapy drugs and immunotherapy drugs may over - activate the immune system, resulting in more severe immune - related adverse reactions.

**6. What are the possible benefits of participating in the study?**

1. **Potential Treatment Efficacy**
   1. The study is evaluating a new treatment regimen (HgcPC) for postoperative adjuvant treatment of intrahepatic cholangiocarcinoma (ICC). If the treatment is effective, participants may experience a reduced risk of tumor recurrence, longer disease - free survival, and potentially longer overall survival.
   2. The combination of hepatic arterial infusion chemotherapy (HAIC) with gemcitabine - cisplatin, PDL1 inhibitors, and capecitabine is designed to target cancer cells more effectively, based on existing knowledge of the mechanisms of these drugs.
2. **Close Medical Monitoring**
   1. Participants will receive close medical supervision throughout the study. This includes regular check - ups, imaging tests, and laboratory evaluations to monitor their health status and the progress of their disease. Any adverse events or side effects will be promptly identified and managed by the medical team.
3. **Contribution to Medical Knowledge**
   1. By participating in the study, patients have the opportunity to contribute to the advancement of medical knowledge and potentially help future ICC patients. Their participation can lead to a better understanding of the disease and the development of more effective treatment strategies.

**7. Are there any costs？**

There are no costs for the patients participating in this study other than those associated with normal medical diagnosis and treatment. In case of any injury related to the study, corresponding treatment and compensation will be provided according to relevant national regulations.

**8. Is personal information confidential?**

Your study data will be stored in West China Hospital of Sichuan University. Researchers, research supervision departments and the Ethics Review Committee may access your medical records. Any public reports regarding the results of this study will not disclose your personal identity. We will make every effort to protect the privacy of your personal medical data and personal information within the scope permitted by law.

**9. Do I have to take part in the study?**

Participation in this study is completely voluntary. You can decline to participate in the study or withdraw from it at any stage without facing discrimination or retaliation. Your medical treatment and rights will not be affected. If you decide to withdraw from this study, please contact your doctor so that your disease can be properly diagnosed and treated.

**Subjects’ statement:** I have read the above introduction to this study and my

researchers have fully explained to me the purpose of this study, the operational

procedure and the possible risks and potential benefits of participating in this study

and answered all my relevant questions.

Consent□ or Refusal□ studies other than this study utilize my research materials

and biological specimens.

**Signature of participant:** Date：

**Signature of participant’s family:** Date：

**Phone number:**

**Doctor's statement:** I have given relevant details of this study to the above

volunteers who participated in this study and provided him / her with an original copy

of a signed informed consent form. I confirm that the circumstances of this study have

been explained to the subjects in detail, in particular, the ethical principles and requirements such as possible risks and benefits, free and compensation, damages and

compensation, voluntary and confidentiality.

**Signature of doctor:**  Date：

**Phone number:**

**Biomedical ethics review board of West China Hospital, Sichuan University**

**Phone number：028-85422654，028-85423237**

肝动脉灌注化疗序贯卡培他滨联合PDL1抑制剂作为肝内胆管癌术后的辅助治疗：一项多中心II期临床研究

尊敬的受试者

我们邀请您参加四川大学华西医院批准开展的“肝动脉灌注化疗序贯卡培他滨联合PDL1抑制剂作为肝内胆管癌术后的辅助治疗：一项多中心II期临床研究”课题研究。本研究估计将有32名受试者自愿参加。本研究已经得到四川大学华西医院生物医学伦理审查委员会的审查和批准。

1. 为什么要开展本项研究？

肝内胆管癌（Intrahepatic Cholangiocarcinoma,ICC）起源于肝胆管内的上皮细胞，是一种致命的恶性肿瘤，5年生存率仅为9%左右。它是第二常见的原发性肝癌，约占原发性肝癌的10%-20%和所有胃肠道恶性肿瘤的3%。此外，ICC的发病率在全球范围内仍呈显着上升趋势。例如,在过去四十年中,美国ICC的发病率增加了140%以上。目前,手术切除仍然是治愈性治疗的主要选择。然而，只有20%-30%的患者在诊断时适合行手术切除[4]。事实上,即使是接受根治性切除的ICC患者,其5年总生存率(overall survival,OS)也仅为20-35%。而术后复发率较高（3年复发率高达80%）是长期预后不佳的主要原因。因此，迫切需要术后辅助治疗来消除“微小残留病灶”以预防术后复发。

目前，指南推荐使用卡培他滨和替吉奥作为胆道癌(Biliary Tract Cancer,BTC)术后的辅助治疗。目前，已有研究证明，PD-1和PDL-1免疫检查点抑制剂联合化疗在治疗晚期BTC中是有效且安全的。此外,肝动脉灌注化疗(Hepatic Artery Infusion Chemotherapy,HAIC)作为一种可以增强抗肿瘤活性同时减少全身化疗副作用的治疗方式，已被证实与系统性治疗联合使用时可以改善晚期BTC患者的预后。虽然上述研究纳入了ICC患者，但实际上，目前还没有研究仅关注ICC患者的术后辅助治疗。这些研究结论是否也适用于ICC患者，仍需通过高质量的研究来证实。因此，本研究设计了一项前瞻性单臂II期研究，以填补ICC术后辅助治疗领域的研究空白。旨在评估肝动脉灌注化疗（吉西他滨+顺铂，GC）序贯卡培他滨联合PDL-1抑制剂（简称HgcCP）作为ICC患者术后辅助治疗的疗效和安全性。

1. 如果参加研究，您需要做什么？

您将遵循以下治疗方案：先进行两个周期的 HAIC_GC，然后接受六个周期的卡培他滨治疗。同时给与8 个周期PDL1抑制剂，具体方案如下：

a)肝动脉灌注化疗（HAIC）联合吉西他滨（GC）

方案：第一天，给予顺铂25mg/m²+吉西他滨1000mg/m²，经动脉灌注半小时。每3周重复1次，共进行2个周期。

b)卡培他滨

方案：1250mg/m²，口服，每日2次，连续服用14天。一个周期为21天，总共6个周期。

c)PDL1抑制剂

方案：按照相应药物的说明书进行8个周期的治疗。

此外，您将根据下表1进行登记、治疗和随访。

表1 入组患者的临床评估时间表

| 评估 | 基线：入组前1周内 | 每周期第1天^c^ | 八周期治疗结束（在最后一次给予PDL1抑制剂后的4周内） | 入组后随访：第一年  每3月1次 | 入组后随访：第2~3年  每6个月一次 |
| --- | --- | --- | --- | --- | --- |
| 入组：资格筛查^a^和知情同意^b^ | x |  |  |  |  |
| 基线患者特征^#^ | x |  |  |  |  |
| 病理评估^#^ | x |  |  |  |  |
| 实验室检查^#^ | x | x | x | x | x |
| 影像学检查^#^ | x | x | x | x | x |
| 毒性/不良事件监测 |  | x | x | x | x |
| 生活质量 | x | x | x | x | x |
| 复发 |  | x | x | x | x |
| 生存 |  | x | x | x | x |

3. 可供选择的诊疗方案有哪些？

目前指南推荐卡培他滨或替吉奥作为BTC患者的术后辅助治疗。因此，您可以选择卡培他滨或替替吉奥进行治疗。

4. 哪些人不宜参加研究?

1. 在进入试验 5 年内有已知的其他恶性肿瘤病史，但已充分治疗的宫颈原位癌或非黑色素瘤皮肤癌除外
2. 因对造影剂过敏而禁忌使用碘剂和钆剂
3. 肝动脉异常导致肝动脉灌注化疗（HAIC）无法实施
4. 孕妇或哺乳期妇女
5. 既往接受过免疫治疗，如抗 PD - 1、抗 PD - L1抑制剂治疗
6. 入组前 3 个月内有胃肠道出血病史
7. 入组前 6 个月内有动脉和静脉血栓事件病史，如心肌梗死、脑血管意外（包括短暂性脑缺血发作、脑出血、脑梗死）、深静脉血栓形成和肺栓塞
8. 有精神类药物滥用或药物滥用史
9. 既往接受过器官移植
10. 有遗传性或获得性出血及血栓倾向，如血友病、凝血功能障碍、血小板减少症、脾功能亢进等
11. 对相关药物过敏
12. 既往曾针对 ICC 进行过任何化疗或免疫治疗
13. 有任何可能干扰方案治疗的严重未控制的疾病

5. 参加研究有哪些风险？

1. 药物不良反应
2. 研究中使用的化疗药物（如吉西他滨-顺铂、卡培他滨）可能会引起多种不良反应。例如，吉西他滨和顺铂可能会引起恶心、呕吐、脱发、骨髓抑制（包括白细胞减少、血小板减少、贫血等），这可能会影响患者的生活质量。在严重的情况下，可能会导致感染和出血等并发症，甚至危及生命。卡培他滨可能会引起手足综合征、胃肠反应和血液毒性。
3. PDL1抑制剂可能会引发免疫相关的不良事件，例如免疫相关的肺炎、免疫相关的肝炎、甲状腺功能障碍、皮疹、腹泻等。这些不良反应可能需要密切监测和及时处理，严重的免疫相关不良事件可能对患者的健康构成严重威胁。
4. 与HAIC相关的风险

HAIC操作本身具有一定的风险，比如可能发生的肝动脉损伤、出血、感染等并发症。此外，HAIC可能会影响肝脏的正常血液供应，导致肝功能受损甚至肝衰竭。

1. 联合治疗的潜在风险

由于本研究中多种药物和治疗方法的联合使用，不良反应的发生率和严重程度可能会增加。例如，化疗药物和免疫治疗药物的联合使用可能会过度激活免疫系统，导致更严重的免疫相关不良反应。

1. 参加研究有哪些可能的好处？

参加本项研究，您的预后有可能获得改善，本项研究还有助于确定哪种治疗方法可以更安全有效地治疗与您具有相似病情的其他病人。

1. 潜在的治疗效果
2. 该研究正在评估一种用于肝内胆管癌（ICC）术后辅助治疗的新治疗方案（HgcCP）。如果治疗有效，参与者可能会降低肿瘤复发的风险，延长无病生存期，并可能延长总生存期。
3. 肝动脉灌注化疗（HAIC）与吉西他滨-顺铂、PDL1抑制剂和卡培他滨的联合使用，旨在根据这些药物作用机制的现有知识，更有效地针对癌细胞。
4. 密切的医疗监测

在整个研究过程中，参与者将接受密切的医疗监督。这包括定期检查、影像学检查和实验室评估，以监测他们的健康状况和疾病的进展。医疗团队将及时识别和管理任何不良事件或副作用。

1. 对医学知识的贡献

通过参与这项研究，患者有机会为医学知识的进步做出贡献，并有可能帮助未来的ICC患者。您们的参与有助于人类对该疾病的更好理解并制定更有效的治疗策略。

1. 参加研究需要支付有关费用吗？

参与本研究的患者除了与正常医疗诊断和治疗相关的费用外，无需承担其他费用。如果发生与研究相关的任何伤害，将根据相关国家规定提供相应的治疗和补偿。

1. 个人信息是保密的吗？

您的研究资料将保存在四川大学华西医院，研究者、研究主管部门、伦理审查委员会可查阅您的医疗记录。任何有关本项研究结果的公开报告将不会披露您的个人身份。我们将在法律允许的范围内，尽一切努力保护您个人医疗资料的隐私和个人信息。

1. 我必须参加研究吗？

参加本项研究是完全自愿的，您可以拒绝参加研究，或在试验的任何阶段随时退出本研究而不会受到歧视和报复，其医疗待遇与权益不受影响。如果您决定退出本研究，请与您的医生联系，以便妥善诊疗疾病。

受试者声明：我已经阅读了上述有关本研究的介绍，我的研究人员已向我充分解释和说明了本研究的目的、操作过程以及参加本研究可能存在的风险和潜在的获益，并回答了我所有相关问题。自愿参加本研究。

我同意□ 或拒绝□ 除本研究以外的其他研究利用我的研究资料和生物标本。

受试者正楷姓名：

受试者签名： 　　 日期：＿ ＿ ＿ ＿ 年 ＿ ＿ 月 ＿ ＿ 日

受试者的联系电话： 　　手机号：

法定代理人正楷姓名： （如适用）

与受试者关系：

法定代理人签名： 日期：＿ ＿ ＿ ＿ 年 ＿ ＿ 月 ＿ ＿ 日

需法定代理人签署的原因：

见证人正楷姓名： （如适用）

见证人签名： 日期：＿ ＿ ＿ ＿ 年 ＿ ＿ 月 ＿ ＿ 日

需见证人签署的原因：

医生声明：我已对上述参加本研究的自愿者说明了该项研究的有关细节，并且为他/她提供一份签署过的知情同意书的原件。我确认已向受试者详细解释了本研究的情况，特别是参加本研究可能产生的风险与受益、免费与补偿、损害与赔偿、自愿与保密等伦理原则和要求。

医生签名： 　 日期：＿ ＿ ＿ ＿ 年 ＿ ＿ 月 ＿ ＿ 日

医生的联系电话：

**四川大学华西医院生物医学伦理审查委员会**  **联系电话：028-85422654，028-85423237**
