# Supplementary material for: Postoperative adjuvant hepatic arterial infusion chemotherapy with gemcitabine-cisplatin sequential capecitabine combined with PDL1 inhibitors in resected high-risk intrahepatic cholangiocarcinom: study protocol for a prospective, multicenter, single-arm, phase 2 trial (HgcCP trial)
Source: Front Oncol. 2025 Jul 30;15:1584007. doi: 10.3389/fonc.2025.1584007 (PMC12343247; doi:10.3389/fonc.2025.1584007)

**1. Standardized AE Grading Workflow**

All AEs will be assessed using a **centralized three-step protocol**:

| **Step** | **Process** | **Timeline** |
| --- | --- | --- |
| **1. Site-Level Assessment** | Clinical investigators grade AEs using CTCAE 5.0 criteria during biweekly visits | Day 0–30 post-treatment |
| **2. Independent Adjudication** | Suspected ≥Grade 3 AEs reviewed by blinded **Toxicity Review Committee (TRC)** | ≤72 hours of initial report |
| **3. DSMB Oversight** | All Grade 4–5 AEs audited by Data Safety Monitoring Board (DSMB) | Quarterly reviews |

**2. Immunotherapy-Related Adverse Events and Management Strategies**

**Immunotherapy-related adverse events (irAEs)** most commonly include dermatologic toxicities, colitis/diarrhea, hepatic toxicities, and endocrinopathies.

**Severe irAEs** are associated with drug class, dose, interval, and treatment duration. Patients experiencing severe toxicity are generally not re-challenged with immune checkpoint inhibitors (ICIs) upon disease progression due to the risk of severe reactions.

**Non-severe irAEs** may allow for ICI rechallenge after temporary discontinuation. Potential biomarkers for irAEs include non-specific markers (e.g., elevated IL-6, associated with poor treatment response) and organ-specific markers (e.g., elevated sCTLA-4 predicting anti-CTLA-4 efficacy and irAE risk).

**(1). Fatigue**

In monotherapy studies, fatigue may be attributable to **hypothyroidism**. Physical activity may alleviate such symptoms.

**(2). Dermatologic Toxicities**

Common skin toxicities include **erythema, rash (maculopapular, pustular), pruritus, and reactive capillary hyperplasia**. Management:

- Grade 1–2: Topical emollients, antihistamines, high-potency corticosteroid creams
- Severe: Intravenous corticosteroids

**(3). Endocrine Toxicities**

Target organs include the **thyroid, pituitary, adrenal glands, and pancreatic β-cells**. Thyroid dysfunction (hypo- or hyperthyroidism, thyroiditis) is most common, typically presenting within 6 months of ICI initiation.

- **Hyperthyroidism**: Withhold ICIs; initiate β-blockers (e.g., propranolol, atenolol, metoprolol). Resume ICIs when asymptomatic.
- **Hypothyroidism**: Initiate levothyroxine (1.6 µg/kg/day), titrate to normalize TSH.
- **Thyroiditis**: Oral prednisone with gradual taper.

**(4). Hepatotoxicity**

Presents as **elevated ALT/AST ± hyperbilirubinemia**, typically **8–12 weeks** after first ICI dose. Liver biopsy may guide management.

| **Grade** | **Management** |
| --- | --- |
| 1–2 | Prednisone 0.5–1 mg/kg if no improvement after 1 week; taper over weeks |
| 3 | **Discontinue ICIs**, start prednisone; add mycophenolate mofetil if no improvement in 2–3 days. Avoid ICI rechallenge. |
| 4 | **Permanently discontinue ICIs**, IV methylprednisolone 2 mg/kg; add mycophenolate if no improvement in 2–3 days. |

**(5). Gastrointestinal Toxicities**

Manifest as **diarrhea/colitis**, typically after **3 ICI doses**. Endoscopy confirms colitis.

| **Grade** | **Management** |
| --- | --- |
| 1 | Continue ICIs; loperamide. |
| 2 | **Hold ICIs**, oral prednisone 1 mg/kg or budesonide; if no improvement in 3–5 days, perform colonoscopy and consider infliximab 5 mg/kg. |
| 3–4 | **Permanently discontinue ICIs**, infliximab; colectomy if perforation occurs. |

**(6). Pneumonitis**

Symptoms: **dry cough, progressive dyspnea, fine inspiratory crackles**. Diagnose via **CT** and bronchoscopy.

| **Grade** | **Management** |
| --- | --- |
| 1–2 | **Hold ICIs**, prednisone 1–2 mg/kg/day; taper over 4–6 weeks if improved. |
| 3–4 | **Permanently discontinue ICIs**, IV methylprednisolone 2 mg/kg/day; add infliximab, mycophenolate, or IVIG if refractory. |

**(7). Cardiotoxicity (Myocarditis)**

Management: Discontinue ICIs, supportive care, and prednisone 0.5–2.0 mg/kg (or IV methylprednisolone 1 g). Refractory cases may require mycophenolate, tacrolimus, or abatacept.

**(8). Infusion Reactions**

- **Mild/moderate**: Slow infusion rate, antihistamines.
- **Severe**: **Permanently discontinue ICIs**, follow anaphylaxis protocols:
  - **Epinephrine**: 0.01 mg/kg IM (max 0.5 mL of 1 mg/mL), repeat q5–15 min.
  - **IV fluids**: 1–2 L crystalloid bolus over 5 min.
  - **Antihistamines**: Diphenhydramine 25–50 mg IV + H2 blocker (e.g., ranitidine 50 mg).
  - **Steroids**: Methylprednisolone 1–2 mg/kg q6h to prevent biphasic reactions.

**(9). Gastrointestinal Perforation**

- **Diagnostic Criteria: CT-confirmed free air + systemic inflammation**
- **Action Protocol:**


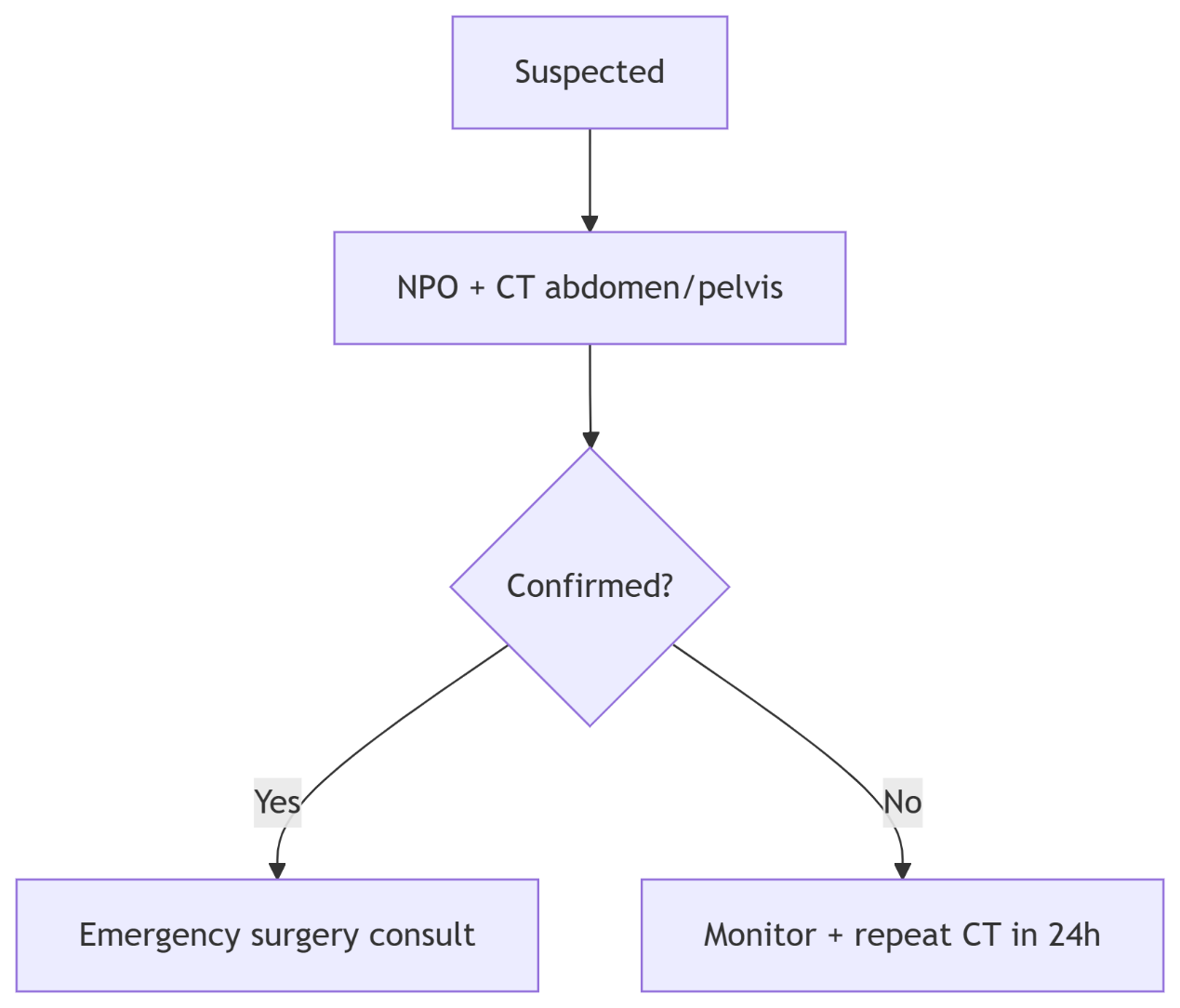

Supplement: Supplementary file 2 [file DataSheet2.docx]
